# Retraction Note: Sulforaphane induces S-phase arrest and apoptosis via p53-dependent manner in gastric cancer cells

**DOI:** 10.1038/s41598-024-82511-7

**Published:** 2024-12-10

**Authors:** Yuan Wang, Huazhang Wu, Nannan Dong, Xu Su, Mingxiu Duan, Yaqin Wei, Jun Wei, Gaofeng Liu, Qingjie Peng, Yunli Zhao

**Affiliations:** 1 School of Public Health, Bengbu Medical College, Bengbu, 233030 People’s Republic of China; 2 School of Life Science, Anhui Province Key Laboratory of Translational Cancer Research, Bengbu Medical College, Bengbu, 233030 People’s Republic of China; 3 School of Clinical Medicine, Bengbu Medical College, Bengbu, 233030 People’s Republic of China; 4 Department of Gastroenterology, The First Affiliated Hospital of Bengbu Medical College, Bengbu, 233030 Anhui People’s Republic of China

Retraction of: *Scientific Reports* 10.1038/s41598-021-81815-2, published online 28 January 2021

The Editors have retracted this Article. After publication, concerns were raised regarding the data presented in this Article. Specifically, Fig. 4A BGC-823 0 μM and MGC-803 5 μM images appear to overlap. The authors have been able to provide the underlying raw data and stated that BGC 0 and 10 μM images were selected incorrectly during figure preparation.

However, the BGC-823 and MGC-803 cell lines used in this study have been reported to be contaminated with HeLa cervical cancer cells, making them unsuitable as a model of gastric cancer.

The Editors therefore no longer have confidence in the results and conclusions of this study.

Huazhang Wu has stated on behalf of all authors that they agree with this retraction, but not the wording of this retraction notice.

